# Inhibition of Protein Kinase CK2 Affects Thymidylate Synthesis Cycle Enzyme Level and Distribution in Human Cancer Cells

**DOI:** 10.3389/fmolb.2022.847829

**Published:** 2022-02-25

**Authors:** Patrycja Wińska, Łukasz Widło, Elżbieta Senkara, Mirosława Koronkiewicz, Jarosław M. Cieśla, Alicja Krzyśko, Katarzyna Skierka, Joanna Cieśla

**Affiliations:** ^1^ Chair of Drug and Cosmetics Biotechnology, Faculty of Chemistry, Warsaw University of Technology, Warsaw, Poland; ^2^ Department of Biomedical Research, National Medicines Institute, Warsaw, Poland; ^3^ Institute of Biochemistry and Biophysics, Polish Academy of Sciences, Warsaw, Poland

**Keywords:** protein kinase CK2, CX-4945, thymidylate synthase, dihydrofolate reductase, serine hydroxymethyltransferase, acute lymphoblastic leukemia cells CCRF-CEM, protein–protein interaction

## Abstract

Thymidylate synthase (TS), dihydrofolate reductase (DHFR), and serine hydroxymethyltransferase (SHMT) constitute the thymidylate synthesis cycle providing thymidylate for DNA synthesis and repair. Our previous studies indicated that TS and DHFR are the substrates of protein kinase CK2. This work has been aimed at the elucidation of the effect of CK2 activity on cell cycle progression, thymidylate synthesis enzyme expression and localization, and the role of CK2-mediated TS phosphorylation in *in vitro* di- and trimolecular complex formation. The results were obtained by means of western blot, confocal microscopy, flow cytometry, quantitative polymerase chain reaction (QPCR), quartz crystal microbalance with dissipation monitoring (QCM-D), and microthermophoresis (MST). Our research indicates that CK2 inhibition does not change the levels of the transcripts; however, it affects the protein levels of DHFR and TS in both tested cell lines, i.e., A549 and CCRF-CEM, and the level of SHMT1 in CCRF-CEM cells. Moreover, we show that CK2-mediated phosphorylation of TS enables the protein (pTS) interaction with SHMT1 and leads to the stability of the tri-complex containing SHMT1, DHFR, and pTS. Our results suggest an important regulatory role of CK2-mediated phosphorylation for inter- and intracellular protein level of enzymes involved in the thymidylate biosynthesis cycle.

## Introduction

CK2 (formerly known as casein kinase II) is a protein kinase involved in regulation of many processes such as transcription (Lüscher et al., 1990; Cabrejos et al., 2004) and translation (Riera et al., 1999; Szebeni et al., 2003; Borgo et al., 2015; Gandin et al., 2016), control of protein stability (Zhang et al., 2002; Patsoukis et al., 2013; Niechi et al., 2015) and degradation (Shen et al., 2001; Scaglioni et al., 2009), cell cycle progression (Homma and Homma, 2008), cell survival (Ahmed et al., 2002; Piazza et al., 2006; Duncan et al., 2011), and circadian rhythms (Tsuchiya et al., 2009). The role of this conserved, constitutively active serine–threonine kinase in a cell regulatory network is highly complex, and the extensive interplay between CK2-mediated phosphorylation and other post-translational modifications has been suggested (Nuñez de Villavicencio–Diaz et al., 2017). Moreover, an increased level/activity of CK2 kinase has been observed in several tumor types (Trembley et al., 2009; Borgo and Ruzzene, 2021).

Most of the CK2 substrates play important roles in the regulation of metabolic processes, cell signaling or apoptosis, including protecting the integrity of DNA (Chapman and Jackson, 2008; Siddiqui–Jain et al., 2012). Among those substrates are also three enzymes, namely, thymidylate synthase (TS), dihydrofolate reductase (DHFR), and serine hydroxymethyltransferase (SHMT), involved in the metabolic cycle resulting in *de novo* synthesis of thymidylate, a crucial substrate for DNA synthesis and repair. The *in vitro* CK2-mediated phosphorylation of TS was shown by Frączyk et al. (Frączyk et al., 2010; Frączyk et al., 2015) and Ludwiczak et al., 2016. The phosphorylation lowered TS catalytic activity and affected binding of the TS inhibitor, 5-fluorodeoxyuridylate (FdUMP). The results of molecular dynamics simulations have shown that CK2-mediated phosphorylation of serine 124 residue of the human TS leads to a protein conformational change, resulting in an unfavorable position of the substrate (dUMP) and cofactor (methylene-THF) in the active center (Jarmuła et al., 2010). In addition, a stiffening of certain protein fragments, especially the loop closing the active center pocket, has been shown (Frączyk et al., 2010). High-resolution structures provided evidence that the native human TS can have two major conformations: active and inactive, which depends on the location of the loop 182–297 (Lovelace et al., 2009). Mutant TS, M190K, with loop 182–297 stabilized in inactive conformation, was highly phosphorylated by CK2 in contrast to the active-conformation-stabilized mutant R163K (Luo et al., 2011). This may indicate the physiological relevance for the conformational switching of TS activity with possible stabilization of the inactive form by phosphorylation. The *in vitro* phosphorylation by CK2 of the second enzyme of the thymidylate synthesis cycle, DHFR, has been demonstrated recently by us (Skierka et al., 2019), while the third enzyme of the cycle, SHMT, has been indicated as the CK2 substrate in phosphoproteomics studies on mitotic HEK 293T cells (Rusin et al., 2017).

Both TS and DHFR are well-known molecular targets in anti-cancer chemotherapy, whereas CK2 has recently emerged as a target (Borgo et al., 2021), and the enzyme inhibitor, CX-4945 (5-(3-chlorophenylamino)benzo [c][2,6]naphthyridine-8-carboxylic acid), also known as silmitasertib (Pierre et al., 2011; Kim and Kim, 2012; Chon et al., 2015) undergoes phase I and phase II clinical studies for cancer treatment. This small molecule, ATP-competitive inhibitor, demonstrates an excellent profile of selectivity. Testing CX-4945 at 500 nM against a panel of 235 kinases revealed that this compound is able to affect the activity of only 49 kinases to an extent greater than 50% (D’Amore et al., 2020). It shows efficacy both *in vitro* in cells and *in vivo* in animal models and has a suitable pharmacokinetic profile (long half-life, oral bioavailability, and no toxicity). CX-4945 has been found to show an extensive anti-proliferative activity, i.e., to promote cell cycle arrest, and to induce caspase activity and apoptosis in various cancer cell lines (D’Amore et al., 2020).

Taking into account that CK2 regulates many oncogenic pathways and processes, we have recently undertaken the studies on simultaneous treatment of cancer cells with inhibitors of TS and CK2 (Wińska et al., 2018; Wińska et al., 2020) or DHFR and CK2 (Wińska et al., 2019) searching for the synergistic effect. The obtained results demonstrate the ability of CK2 inhibitors to enhance the efficacy of 5-fluorouracil (FU, TS inhibitor) or methotrexate (MTX, DHFR inhibitor) in cancer cells. The molecular mechanism of the observed synergistic effect that occurred in MCF-7 cells after CX-4945 and FU simultaneous treatment was not clear; however, the synergistic effect seemed to be related to the delay of FU-induced S-phase arrest recovery. In the MTX and CX-4945 case, we have observed cell accumulation in both the S-phase and G2/M-phase of the cell cycle after the combined treatment of CCRF-CEM cells, which seems to result from an additive effect, since MTX and CX-4945 used separately led to G2/M-phase arrest and S-phase arrest, respectively. Additionally, we have observed a significant increase in the DHFR level in acute lymphoblastic leukemia cells (CCRF-CEM) after treatment with CX-4945 (Wińska et al., 2019), which might indicate the possible involvement of CK2 in the regulation of thymidylate synthesis and, therefore, may affect CX-4945 therapy outcome.

In this follow-up study, we investigate the effect of CK2 inhibition on DHFR and TS expression in two different cancer cell lines, A549 (adenocarcinomic human alveolar basal epithelial cells) and CCRF-CEM (human acute lymphoblastic leukemia cells), while the expression of two forms of serine hydroxymethyltransferase, cytosolic (SHMT1) and mitochondrial (SHMT2), was studied in the CCRF-CEM cell line. The effect of CX-4945 on cell cycle progression and DHFR and TS localization has been investigated in A-549 cells. Additionally, the effect of CK2-mediated TS phosphorylation on the formation of di- and trimolecular complexes with SHMT1 and DHFR was studied by means of quartz crystal microbalance with dissipation monitoring (QCM-D) and microthermophoresis (MST). The obtained results suggest an important regulatory role of CK2-mediated phosphorylation in the stability of SHMT/DHFR/TS in cells, cellular localization of DHFR and TS, and the ability of the three enzymes to interact with each other.

## Materials and Methods

### Reagents and Antibodies

Dimethyl sulphoxide (DMSO), molecular biology grade, used as a solvent for all stocks of chemical agents, was obtained from Roth (Karlsruhe, Germany). All reagents used in flow cytometry analysis were purchased from BD Biosciences Pharmingen (San Diego, CA, United States). The following primary antibodies were used: anti-GAPDH (Merck Millipore, #MAB374, 1:20000, 30 min, RT), anti-p-p65 (Ser529) (Biorbyt, # orb 14916, 1:500, overnight, +4°C), anti-DHFR (BD Biosciences), and anti-TS (Merck Millipore, #MAB4130, 1:500, overnight, +4°C). Secondary goat anti-rabbit IgG-HRP (Dako, #P0448, 1:2000, 1h, RT) and anti-mouse IgG-HRP (Dako, #P0447, 1:1000, 1h, RT) were used. Hoechst 33342 (Life Technologies, #H3569) and anti-mouse Alexa Fluor 555 were used in the IF study. Protease inhibitors (#11 836 153 001) were from Roche Applied Science (Mannheim Germany). The nitrocellulose membrane was from GE Healthcare Life Sciences (Freiburg, Germany), solvents for HRP reaction (Western Bright Peroxide and Western Bright Quantum) were purchased from Advansta, ECL reagent was from Millipore (United States), and CX-4945 was obtained from Biorbyt. Other solvents, reagents, and chemicals were purchased from POCH (Avantor Performance Materials, Gliwice, Poland), Merck, and Sigma-Aldrich Chemical Company (St. Louis, MO, United States).

### Cell Culture and Treatment of Agents

CCRF-CEM (ECACC 85112105) human Caucasian acute lymphoblastic leukaemia was purchased from ECACC, whereas A-549 (ATCC CCL-185) human lung carcinoma was purchased from ATCC. A-549 cell line was cultured in high-glucose DMEM (Lonza, Basel, Switzerland) supplemented with 10% fetal bovine serum (EuroClone), 2 mM l-glutamine, and antibiotics (100 U/ml penicillin and 100 µg/ml streptomycin). CCRF-CEM was cultured in RPMI 1640 supplemented with 10% fetal bovine serum (EuroClone), 2 mM l-glutamine, and antibiotics (100 U/ml penicillin and 100 µg/ml streptomycin). Cells were grown in 75 cm^2^ cell culture flasks (Sarstedt, Nümbrecht, Germany), in a humidified atmosphere of CO_2_/air (5/95%) at 37°C. All the experiments were performed in exponentially growing cultures. Stock solution of CX-4945 was prepared in DMSO and stored in −80°C for maximum 1 month. For the cytotoxicity studies, the stock solution of CX-4945 was diluted 200-fold with the proper culture medium to obtain the final concentration. For cytotoxicity studies, 2-fold serial dilutions of CX-4945 were prepared in the proper medium containing 0.5% DMSO.

3-(4,5-Dimethylthiazol-2-yl)-2,5-diphenyltetrazolium bromide (MTT)-based viability assay: after incubation with the test compounds, an MTT test was performed as described previously (Wińska et al., 2020). Optical densities were measured at 570 nm using a BioTek microplate reader. All measurements were carried out in a minimum of three replicates.

### Cell Cycle Analysis

A-549 cells were cultured in 25 cm^2^ culture flasks and treated with the tested compounds as it was described above. After exposure to CX-4945, the cells were trypsinized, collected, and washed with cold PBS and fixed at –20°C in 70% ethanol for at least 24 h. Subsequently, the cells were washed in PBS and stained with 50 μg/ml PI (propidium iodide) and 100 μg/ml RNase solution in PBS supplemented with 0.1% v/v Triton X-100 for 30 min in the dark at the RT. Cellular DNA content was determined by flow cytometry employing a CyFlow Cube 8 (Sysmex, Norderstedt, Germany) flow cytometer. The DNA histograms obtained were analyzed using FCS Express 5 Flow software (*De Novo* Software, Glendale, CA, United States) for evaluation of distribution of the cells in different phases of the cell cycle.

### Western Blotting

Adherent cells growing exponentially were seeded at 4.8 × 10^5^ cells in 6 cm diameter plates, whereas CCRF-CEM cells were seeded in a concentration of 2 × 10^5^ cells/ml in 25 cm^2^ flasks (Sarstedt). Subsequently, CX-4945 was added in a final concentration of 0.5% DMSO in concentrations corresponding to 0.5 IC_50_, IC_50_, and 1.5 IC_50_ for adherent cells and 0.5 IC_50_, IC_50_, and 2 IC_50_ for CCRF-CEM. After incubation, the suspension cells were collected by centrifugation at 260 RCF and washed 3 times with ice-cold PBS and supernatants were discarded and pellets were storage at –20°C up to 1 month. In order to obtain lysates, RIPA buffer (50 mM Tris-HCl pH 7.4, 1% NP-40, 0.5% sodium deoxycholate, 0.1% SDS, 150 mM NaCl, 2 mM EDTA, 50 mM NaF, 0.2 mM sodium orthovanadate, and protease inhibitors cocktail, Roche) was added as it was described elsewhere (Wińska et al., 2020). Adherent monolayer cells were washed three times in ice-cold PBS, and the cells were scraped and lysed in RIPA as it was described previously (Wińska et al., 2020). Cellular lysates were analyzed by western blotting as it was described previously (Wińska et al., 2019).

### Densitometry

For densitometry, immunoblots were scanned using G Box Chemi (Syngene), and the density of each lane of phosphorylated and total protein was quantified, using Image J software. Phosphorylated protein densities were normalized to GAPDH densities, assuming 1 for untreated cells, and then, they were converted to a percent of the appropriate control.

### Statistical Evaluation

Results are represented as mean ± s.e.m. of at least three independent experiments performed in triplicate. Statistical analysis was performed using the GraphPad Prism 5.0 software (GraphPad Software Inc., San Diego, CA, United States). Significance was determined using a *t*-test. The statistical significance of differences was indicated in figures by asterisks as follows: *p ≤ 0.05, **p ≤ 0.01, and ***p ≤ 0.001.

### Immunocytochemical Staining and Microscopy Analysis

Cells were seeded in 4-well dishes (35/10 mm; Greiner Bio-One North America, Inc.) and cultured in respective conditions. After 18 h culturing, cells were treated with 0.5% DMSO (control) or 15.5, 31, or 46.6 µM CX-4945 for indicated incubation time. Subsequently, cells were washed twice with PBS, fixed with 3.7% paraformaldehyde solution (PFA) for 20 min, washed twice with PBS, and incubated for 30 min at the room temperature with permeabilization and blocking solution (5% BSA, 0.1% Triton X 100, and 0.5% Tween 20 in PBS). Subsequently, the solution was discarded, and the cells were washed three times with PBST (0.1% Tween 20 in PBS) and incubated with the primary antibodies (anti-DHFR or anti-TS, both 1:100) in PBST for 24 h at 4°C. After washing with PBST (0.5 ml/compartment), the cells were protected from light and incubated with Alexa Fluor 546-conjugated anti-mouse diluted 1:500 (ThermoFisher Scientific, United States) for 1 h at the room temperature. After washing, the cells were incubated in 1 µl/ml Hoechst 33342 in PBST for 15 min. The dye solution was discarded, and the cells were washed with PBST (0.5 ml/compartment). Pictures were taken using the Olympus Fluoview FV1000 confocal laser scanning microscope (CLSM, Olympus, Center Valley, PA, United States).

### Total RNA Isolation and Quantitative Polymerase Chain Reaction

Total RNA isolation was performed with the use of Renozol TRI RNA extraction reagent (GenoPlast Biochemicals), and reverse transcription was conducted using a High Capacity cDNA Reverse Transcription Kit (Applied BioSystemsTM) according to the protocols of manufacturers. The obtained cDNA was a template in quantitative polymerase chain reaction (QPCR), which has been performed virtually as described previously (Wińska et al., 2019). The primers used for the amplification of SHMT1 were 5′ CCT​GTC​CAG​GTG​ACA​GAA​G 3’ (forward) and 5′ TGC​CAG​TCT​CTC​CTT​GAA​C 3’ (reverse) and for SHMT2 amplification were 5′ AGC​TCA​TTG​CCT​CAG​AGA​AC 3’ (forward) and 5′ TGC​AGG​ATC​CAG​GTC​AAA​G 3’ (reverse). The primers were designed using the Kalign tool available at www.ebi.ac.uk (Chojnacki et al., 2017) and ordered from the DNA Sequencing and Oligonucleotides Synthesis Laboratory at the Institute of Biochemistry and Biophysics (Warsaw, Poland). QPCR was performed with the use of Real-Time 2xHS-PCR Master Mix SYBR®A (A&A Biotechnology Gdynia, Poland) as it was previously described (Borkiewicz et al., 2019). The amount of target mRNA was calculated by the ΔCt method (Vandesompele et al., 2002) with the geometric mean of Cts of two reference genes, β-glucuronidase (GUSB, NM_000181) and TATA-binding protein (TBP, NM_003194). Relative SHMT1 and SHMT2 gene expression in control cells (RE) was set at a value of 1, and the relative SHMT1 or SHMT2 gene expression after treatment with the inhibitor (RE + I) was calculated as the ratio RE + I/RE. Values of experimental measurements were considered as independent variables. Rare outlying results were omitted in further calculations. In the next step, the ratio of each relative gene expression in the sample from the cells treated with the inhibitor versus each relative gene expression in the sample from the untreated cells was calculated. This yielded a two-dimensional matrix of values comparing gene expression in untreated and treated cells. If compared expressions are the same, these ratios should equal 1. Therefore, it was possible to apply the chi-square test with 1 as the expected value and the average of all results from the matrix as the observed value. Significantly outlying quantiles from the matrix were omitted in this test. The result of the chi-square test is the probability that the gene expression in treated cells is equal to the gene expression in untreated cells. Expression in both groups of cells was considered different when the probability of this equality was lower than 0.05 (*p* ≤ 0.05).

### Protein Preparations

Recombinant His-tagged DHFR and TS were overexpressed and purified as described previously (Antosiewicz et al., 2015; Skierka et al., 2019). Recombinant SHMT1 was overexpressed and purified with the following modifications. The Ni-NTA binding solution contained 50 mM phosphate buffer pH 7.5, 300 mM NaCl, 10 mM imidazole, 50 μM pirydoxal phosphate (PLP), 2 μg/ml aprotinin, 2 μg/ml leupeptin, and 15 μg/ml PMSF. The column was washed with 50 mM phosphate buffer containing 300 mM NaCl, 50 μM PLP, and 50 mM imidazole and eluted with the same buffer except that 250 mM imidazole was used. The purest fractions were pooled, dialyzed against 20 mM Tris-HCl pH 7.5, 500 mM NaCl, 50 μM PLP, and 10% glycerol, and stored at −20°C.

For His-tag removal, the Thrombin CleanCleave Kit (Sigma-Aldrich) was used according to the manufacturer’s recommendations.

TS was phosphorylated using CK2α enzyme preparation. The reaction was conducted in 4 ml of 20 mM Tris-HCl buffer, pH 7.5 containing 98 μM His-tagged human TS, a 2.8 μM CK2α subunit, 15 mM MgCl2, 280 μM ATP, and 6 mM 2-mercaptoethanol at 30°C for 1 h. Phosphorylated and non-phosphorylated fractions were separated using the method of Wolschin et al. (Wolschin et al., 2005). Fractions containing phosphorylated and non-phosphorylated proteins were adjusted to contain 50 mM phosphate bufor pH 7.6, 300 mM NaCl, and 10 mM imidazole and uploaded onto Ni_NTA columns in order to purify them from CKα’ and concentrate. Eluted His-tagged TS and PTS were dialyzed against 50 mM phosphate buffer pH 7.6 and 10% glycerol.

The protein concentrations in all preparations were assayed using the Bradford method (Bradford, 1976).

### QCM-D Assay

The protein–protein interactions were assayed according to the work of Antosiewicz et al., 2015, using commercial His-tag capturing sensors QSX340, with slight modifications. A His-tagged protein was immobilized on the sensor and served as a receptor, whereas the ligand protein (or proteins) was introduced into the measuring chamber. Before the immobilization of the His-tagged protein, the sensor alone was measured to exclude non-specific adsorption of the tagless ligand protein. Additionally, the binding of the His-tagged ligand to the sensor surface already functionalized with His-tagged receptor was excluded.

For the study of two-protein interactions of SHMT1 with TS, pTS, or DHFR, 5 μM His-tagged SHMT was immobilized on the sensor surface, whereas the tagless ligand protein was introduced into the chamber in various concentrations (in the range of 0.1–1.0 μM or 0.5–7.0 μM in case of TS, pTS, or DHFR, respectively).

For the study of three-protein interactions, 5 μM His-tagged SHMT1 was immobilized on the sensor surface followed by the introduction of mixture containing a fixed concentration of TS or pTS (1.0 μM) and various concentrations of DHFR (in the range of 0.25–2.0 μM).

All measurements were conducted in 20 mM Tris-HCl (pH 7.6) containing 100 mM, 10 mM MgCl_2_, and 0.05% Tween 20 at 25°C and 200 μl/min flow rate.

The Kd values were obtained by fitting the experimental data to the Langmuir isotherm model using QTools software.

The diagram illustrating the QCM-D method is given in Supplementary Figure S1.

### MST Assay

The microthermophoresis assays were optimized and performed as previously described (Antosiewicz et al., 2015) using the MST Monolith.115 instrument, courtesy of NanoTemper Technologies company. Di- and tri-complex formation was monitored using Premium MST capillaries. The diagram of the MST procedure is shown in Supplementary Figure S2. The temperature-induced fluorescent change as a function of the titrant concentrations enables the determination of the strength of interactions occurring. The SHMT–DHFR interaction was measured using fluorescently labeled DHFR protein and various concentrations of SHMT in the range of 157 μM–4.79 nM. For SHMT–TS and SHMT–pTS interactions, either SHMT or pTS was labeled and the other protein was used as a titrant (TS in the range of 62.0 μM–1.9 nM and SHMT in the range of 157 μM–4.79 nM). For tri-complex formation assay, DHFR was labeled and samples containing constant concentrations of labeled DHFR and TS or pTS (9.0 μM) and SHMT titrant concentration in the range of 157 μM–4.79 nM were used. The dissociation constant Kd is obtained by fitting the binding curve with the quadratic solution for the fraction of fluorescent molecules that formed the complex, calculated form the law of mass action. We used DI.Screening Analysis software (NanoTemper) to fit the Kd model which allows to determine the strength of molecular interactions occurring with either 1:1 stoichiometry or where several molecules A bind to one molecule B independently, i.e., with no cooperativity (Supplementary Figure S5).

## Results

### The Effect of CK2 Inhibition on the Level of DHFR, TS, SHMT1, and SHMT2 in Cellular Lysates

**FIGURE 1:**
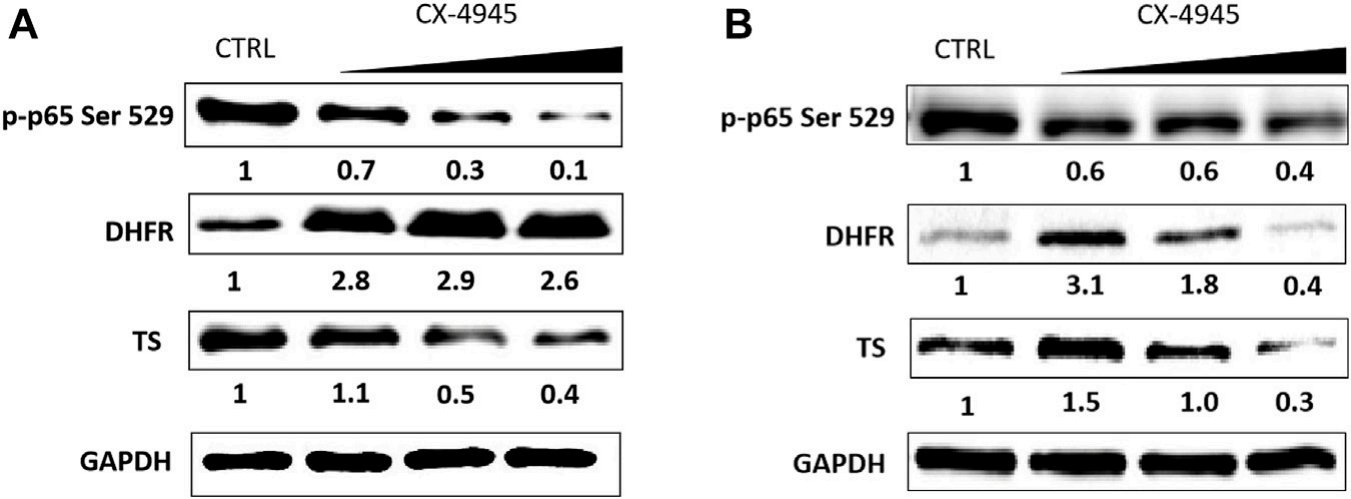
The effect of CX-4945 on the level of p-p65 Ser 529 (nuclear factor kappa-light-chain-enhancer of activated B cells), DHFR, and TS in CCRF-CEM **(A)** and in A-549 **(B)**. Western blot analysis of the following proteins in the crude extracts obtained from the tested cell lines after 48 h of treatment with CX-4945 used in the concentrations of 15.54, 31.08, and 46.62 µM for A-549 and 2, 4, and 8 µM for CCRF-CEM. GAPDH was used as a loading control for each sample. Preparation of cell extracts and protein detection are described in *Materials and Methods*. Densitometry quantifications for each tested protein, given under each cell line panel, were calculated with untreated cells (CTRL) serving as the reference point.

DHFR and TS protein levels were measured by means of western blot in A-549 and CCRF-CEM, whereas the levels of SHMT1 and SHMT2 were measured in CCRF-CEM cells. The cells were treated with the increasing concentrations of CX-4945 within the range from 0.5 IC_50_ to 1.5 IC_50_ (A-549) or 0.5 IC_50_ to 2 IC_50_ (CCRF-CEM) determined after 48 h of incubation. IC_50_ values for CX-4945 are 4 and 31 µM for CCRF-CEM and A-549, respectively (Supplementary Figure S3). To confirm the intracellular inhibition of CK2, we evaluated a site-specific phosphorylation of Ser 529 in p65NF-κB. Dose-dependent CK2 inhibition was obtained in both tested cell lines with the highest efficiency in CCRF-CEM (Figure 1A). The obtained results indicate that inhibition of CK2 affects the level of the tested proteins, but the correlation between the effect and dose of CX-4945 is not simple and depends on a cell line. The relative level of DHFR increases in both tested cell lines up to 3.1 in A-549, with the exception of the highest concentration of CX-4945 (Figure 1B). The relative protein level of TS varies in the range from 0.3 to 1.5 in A-549 and from 0.4 to 1.1 in CCRF-CEM, respectively, at the highest and lowest concentration of CX-4945 (Figure 1).

**FIGURE 2:**
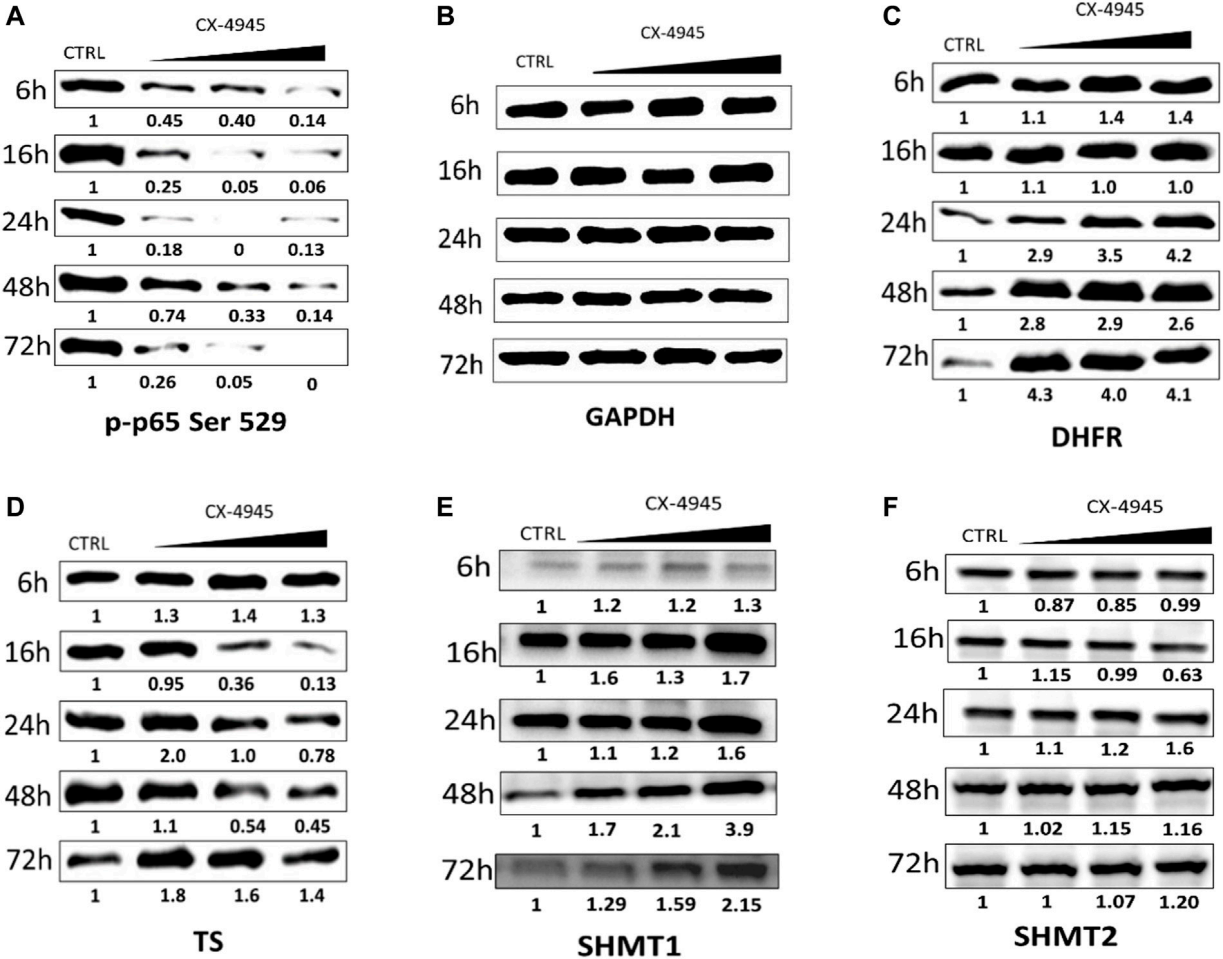
The effect of CX-4945 on the level of p-p65 Ser 529 (nuclear factor kappa-light-chain-enhancer of activated B cells), DHFR, TS, SHMT1, and SHMT2 in CCRF-CEM. Western blot analysis of the following proteins in the crude extracts obtained from CCRF-CEM after 6, 16, 24, 48, and 72 h of treatment with 2, 4, and 8 µM CX-4945: **(A)** p-p65 Ser 529; **(B)** GAPDH was used as a loading control for each sample; **(C)** DHFR; **(D)** TS; **(E)** SHMT1; and **(F)** SHMT2. Preparation of cell extracts and protein detection are described in *Materials and Methods*. Densitometry quantifications for each tested protein were calculated with untreated cells (CTRL) serving as the reference point showing relative protein levels.

In the next step of our investigation, we studied the effect of CK2 inhibition on the relative level of TS, DHFR, and two forms of SHMT, i.e., cytosolic (SHMT1) and mitochondrial (SHMT2) forms, in CCRF-CEM after different times of incubation with CX-4945. The research was conducted after 6, 16, 24, 48, and 72 h of incubation in cells treated with three concentrations of CX-4945, corresponding to 0.5 IC_50_, IC_50_, and 2 IC_50_. An efficient and dose-dependent CK2 inhibition was obtained in CCRF-CEM cells after 6, 16, 48, and 72 h of incubation with CX-4945 (Figure 2A) with the weakest inhibition, i.e., 74% phosphorylation of Ser529 in p65, detected in cells treated with 2 µM CX-4945 for 48 h. GAPDH was used as a loading control for each sample (Figure 2B).

The relative expression of DHFR increases in CCRF-CEM after treatment with CX-4945 up to 4.3 after 72 h of incubation and in a dose-dependent manner after 6 and 24 h of treatment (Figure 2C). The relative protein level of TS increases in lower concentrations of CX-4945 up to 2 after 24 h of incubation, with the exception of 16 h of incubation and decreases in higher doses of CX-4945 with the lowest relative expression of 0.13 after 16 h (Figure 2D). The relative protein levels of SHMT1 and SHMT2 change in a different way with the bigger changes visible for SHMT1 with the relative expression up to 3.9 at the highest concentration of CX-4945 after 48 h of treatment (Figure 2E), whereas the relative level of SHMT2 increases the most after 24 h of treatment up to 2.02 at the highest concentration of CX-4945 (Figure 2F).

### mRNA Levels in CCRF-CEM Cells Treated With CX-4945

**FIGURE 3:**
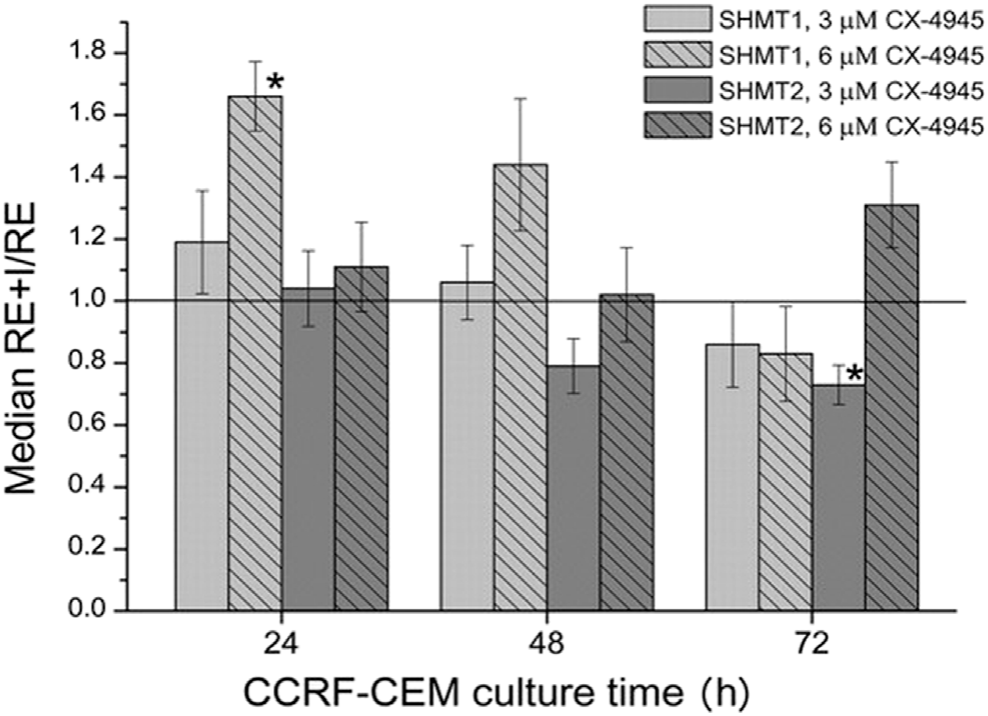
The effect of CX-4945 on the mRNA level of cytosolic (SHMT1) and mitochondrial (SHMT2) serine hydroxymethyltransferases. The cells were treated with 3 μM or 6 μM CX-4945 for 24, 48, and 72 h. The RE + I/RE value equal to 1.0 means that the mRNA level in cells treated with the inhibitor is the same as in untreated cells. The asterisk indicates statistically significant results.

In order to examine whether the observed level of SHMT1 and SHMT2 proteins in CCRF-CEM upon treatment with CX-4945 correlate with the cytosolic SHMT1 and/or mitochondrial SHMT2 transcript levels, QPCR was employed. The SHMT1 and SHMT2 gene expression as the ratio of relative gene expression in cells treated with the inhibitor (RE + I) and the relative gene expression in untreated cells (RE) is shown in Figure 3. The expression of DHFR and TS genes on the mRNA level, measured by QPCR, has been previously described, and the observed changes were insignificant in a majority (Wińska et al., 2019). The results obtained in this study indicate that the changes in SHMT1 and SHMT2 transcript levels upon the treatment with CX-4945 are statistically insignificant, except for the increase in SHMT1 expression in cells treated for 24 h with 6 μM inhibitor and decrease in SHMT2 gene expression in cells treated for 72 h with 3 μM inhibitor. However, the changes are small, below 2-fold in both cases.

SHMT1 mRNA level in CCRF-CEM cells treated for 24 h with 3 μM CX-4045 seems to increase only slightly when compared to untreated cells; however, this level increases more clearly in cells treated with higher (6 μM) CK2 inhibitor concentration (Figure 3). This corresponds to the same trend in the dependence of SHMT1 protein level on CX-4945 concentration (Figure 2E). In case of SHMT2, mRNA level in cells treated with 3 and 6 μM for 24 and 48 h do not change or decrease, whereas protein level slightly increases with the concentration of CK2 inhibitor (comparing Figure 2F, 24 and 48 h, and Figure 3).

### The Effect of CX-4945 Treatment on Localization of DHFR and TS in A-549 Cells

**FIGURE 4:**
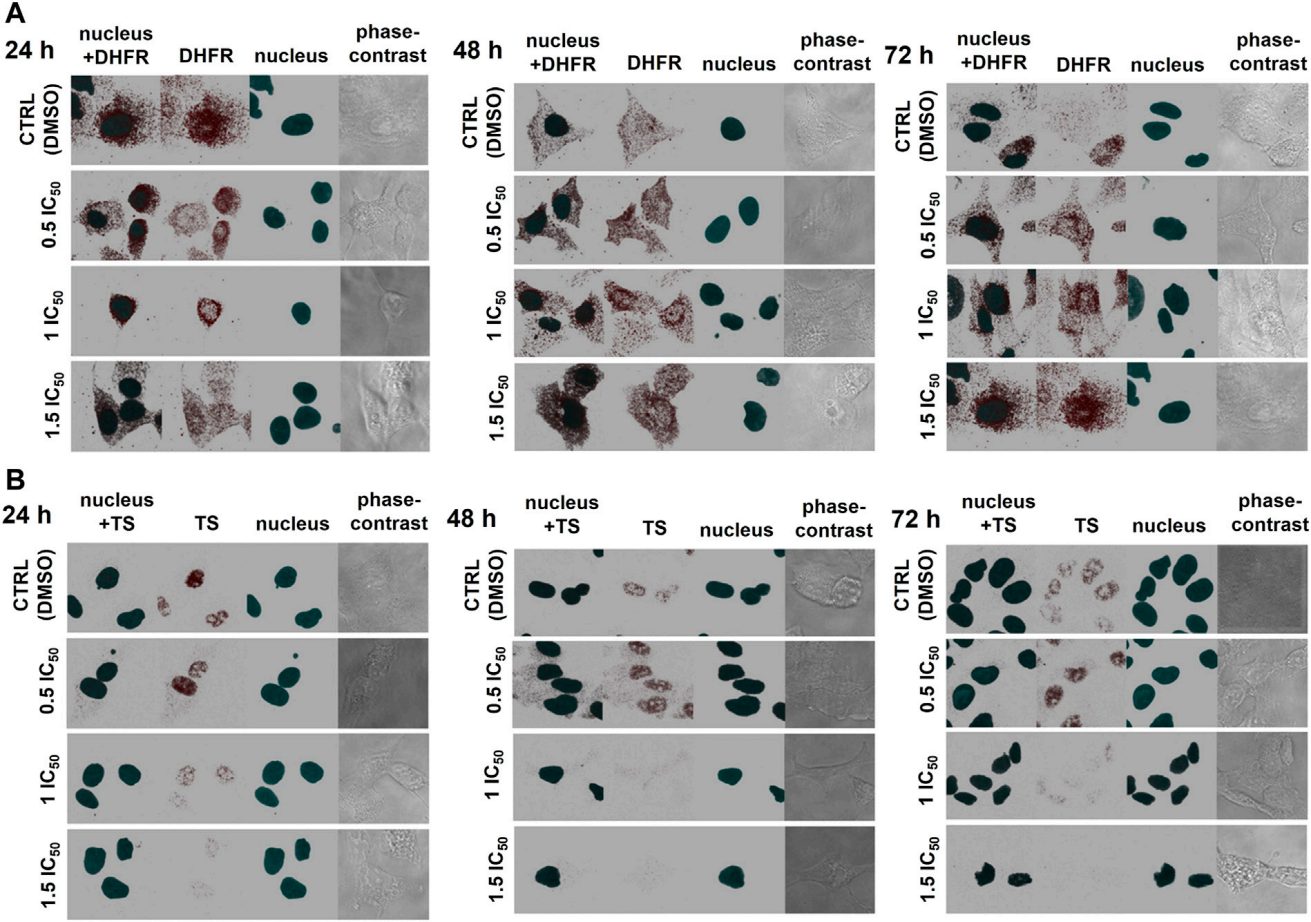
The effect of CX-4945 on the localization and protein levels of DHFR and TS in A-549 cells. The cells were treated with CX-4945 used in concentrations corresponding to 0.5 IC_50_, 1 IC_50_, and 1.5 IC_50_, fixed after incubation for 24, 48, and 72 h; after blocking, they were probed with the primary anti-DHFR **(A)** or anti-TS antibodies **(B)**. Subsequently, the cells were treated with Alexa Fluor 555-conjugated secondary antibody (red fluorescence) and Hoechst 33342 (nuclei, blue fluorescence). The presented pictures of DHFR, TS, nucleus, and combined are 3D pictures of at least 15 z-stack photos visualized together to show the full three-dimensional distribution of the examined proteins. The pictures in the fourth column of each panel are 2D phase contrast photographs. Each picture is shot with the same zoom settings. Photographs represent 65 × 65 µm pictures.

To explain the regulatory role of presumed CK2-mediated phosphorylation of DHFR and TS, we investigated the effect of CK2 inhibition on the distribution and localization of DHFR and TS in A-549 cells. Immunofluorescence was used to detect both enzymes in A-549 cells after 24, 48, and 72 h treatment with CX-4945 used in 15.5 µM (0.5 IC_50_), 31 µM (IC_50_), and 46.5 µM (1.5 IC_50_) concentrations, and the results are shown in Figure 4. The comparison of the investigated thymidylate synthesis cycle enzyme distribution in the untreated lung carcinoma cells shows that DHFR is quite evenly spread in whole cells (in the cytoplasm and nuclei) and stays that way after 24, 48, and 72 h treatment (Figure 4A), while TS is found mainly in the nuclei. Upon treatment with CX-4945, DHFR localization changes with CX-4945 concentration, but it is not a simple correlation. After 24 h treatment, there is a higher immunodetection of DHFR in the nuclei as compared to control cells, especially at 0.5 IC_50_ and 1 IC_50_ concentrations of the CK2 inhibitor. Additionally, the significant increase of DHFR signal is observed in the treated cells after 48 and 72 h (with the exception of the highest concentration of CX-4945) in comparison to the untreated cells. These results correlate with the DHFR protein levels assayed by western blot analysis (Figure 1B). The highest immunodetection of TS, localized mainly in the nuclei, is demonstrated in cells treated with 15.5 µM CX-4945 (0.5 IC_50_) for all incubation times (Figure 4B). The higher inhibitor concentration causes a decrease in TS signal. The immunodetection of TS corresponds to the western blot analysis that shows an increase in TS protein level in A-549 treated with the lowest concentration of CX-4945 and its decrease in cells treated for 48 h with higher concentrations of CX-4945 (Figure 1B).

### The Effect of CX-4945 Treatment on Cell Cycle Progression in A-549 Cells

**FIGURE 5:**
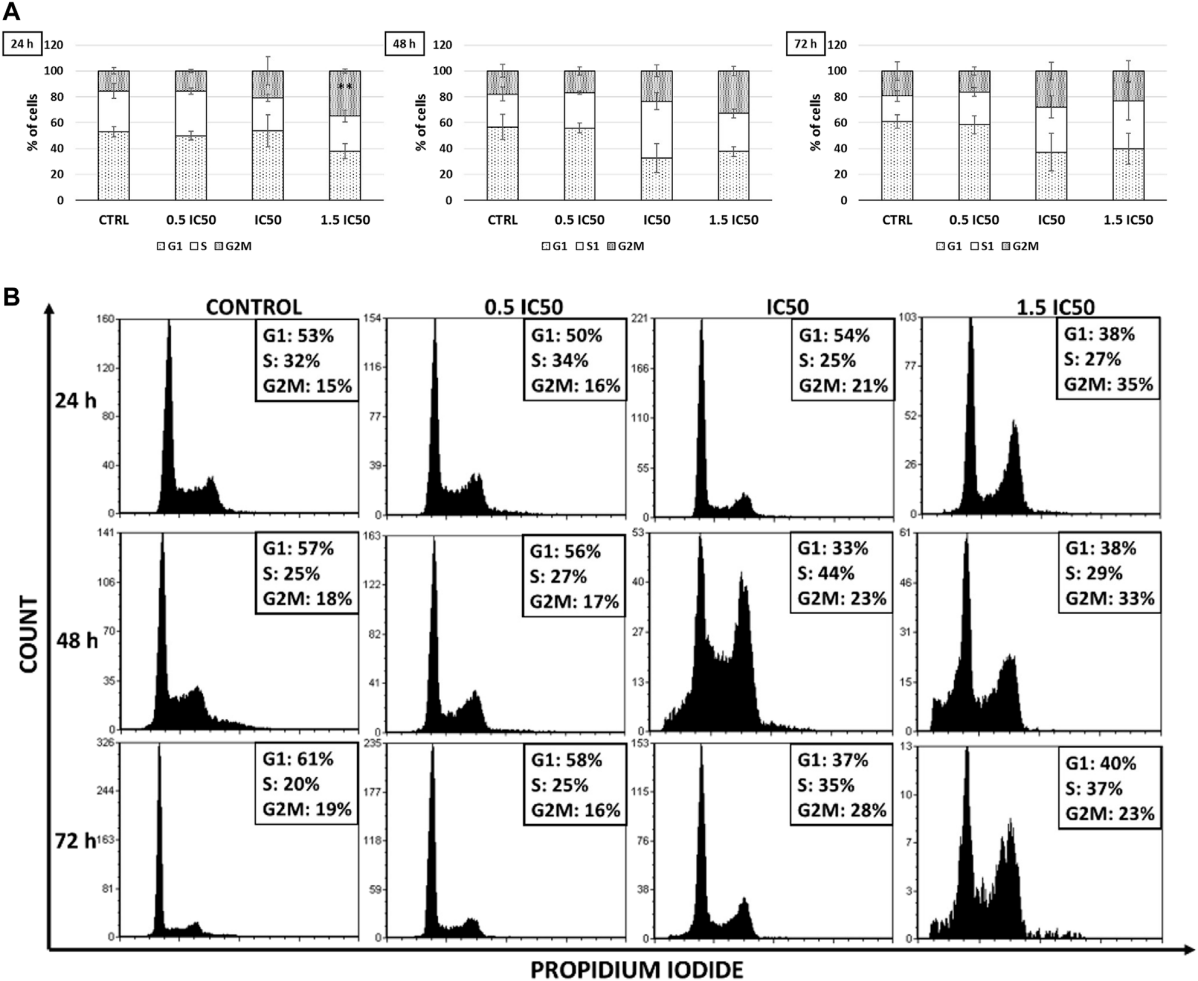
The effect of CX-4945 on cell cycle progression in the A-549 cell line. Cells were treated with 15.5, 31, and 46.5 µM CX-4945 for 24, 48, or 72 h and then fixed and stained with PI. **(A)** Distribution of the cells in different phases of the cell cycle were determined by flow cytometry and analyzed with FCS Express 5 Flow software to determine the percentage of cells in each phase of the cell cycle. Statistically significant results for the cells treated from the control cells by Student’s t-test were indicated by asterisks as follows: *p ≤ 0.05, **p ≤ 0.01, and ***p ≤ 0.001 (see *Materials and Methods*). **(B)** Exemplary DNA histograms of the A-549 cell line.

In order to explain the observed differences in the expression and distribution of DHFR and TS in A-549 cells, the effect of 15.5, 31, and 46.5 µM CX-4945 on cell cycle progression was tested by flow cytometry after 24, 48, and 72 h of treatment. The representative plots with the calculations of cell percentages in each phase of the cell cycle are shown in Figure 5. Distribution of control cells in each phase changes during the cell cycle progression, with the highest difference occurred for the S-phase with 12% less cells in this phase after 72 h of incubation in comparison to 24 h of incubation. Interestingly, the results correspond to confocal microscopy studies that demonstrated the decrease in TS in control nuclei with the time of incubation (Figure 4). Furthermore, the obtained results indicate that CX-4945 leads to prolongation of the G2/M-phase in A-549 cells up to a maximum of 20% more cells in this phase than in control after 24 h of treatment with the highest concentration of CX-4945 (statistically significant result, *p* = 0.004). Additionally, S-phase arrest is detected in the treated cells after 48 and 72 h of treatment with CX-4945. There are up to 19% more cells treated with 31 µM CX-4945 than in control for 48 h.

### The Effect of CK2-Mediated TS Phosphorylation on the Formation of Di- and Tri-Protein Thymidylate Cycle Enzyme Complexes

We have previously described the strong *in vitro* interaction occurring between TS and DHFR proteins (Antosiewicz et al., 2015) and their co-localization in normal and cancer cells (Antosiewicz et al., 2017). In this follow-up study, we show the interactions between the pairs TS and SHMT1, DHFR, and SHMT1 and the formation of the tri-molecular complex consisting of all three thymidylate synthesis proteins, TS, DHFR, and SHMT1. The effect of CK2-mediated TS phosphorylation on the formation and stability of both di- and tri-protein complexes has turned out to be particularly interesting.

**FIGURE 6:**
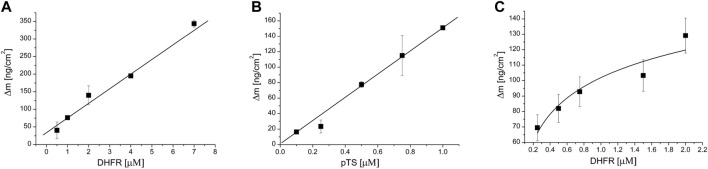
Langmuir isotherm showing the dependence of the weight change on the QCM-D sensor surface on ligand concentration. SHMT was immobilized on the sensor surface, whereas increasing concentrations of indicated thymidylate synthesis cycle enzymes were introduced into the measuring chamber as ligand or ligands. **(A)** SHMT-DHFR interaction, DHFR was a ligand; **(B)** SHMT-pTS interaction, pTS was a ligand; and **(C)** SHMT-DHFR-pTS interaction, fixed pTS and variable DHFR concentrations were used.

Both methods used, QCM-D and MST, showed that the non-phosphorylated TS did not interact with SHMT1 (Table 1, Supplementary Figures S1–S5). However, experiments conducted with CK2α-phosphorylated TS fraction (pTS) confirmed the formation of a strong SHMT1/pTS complex with a dissociation constant Kd = 5.35 ± 0.87 μM obtained from QCM-D studies (Figure 6) and 0.219 ± 0.02 μM) given by MST studies. Thus, in this case, phosphorylation of TS seems to determine the formation of the studied complex. The influence of this post-translational modification of TS molecule is also significant in the case of tri-enzymatic complex formation. Our studies show that although the presence of DHFR enables the formation of SHMT1/DHFR/TS tri-complex, it is weaker than the one formed in the presence of the phosphorylated TS fraction (Figure 6; Supplementary Figure S5). Dissociation constants for di- and tri-protein complexes obtained using QCM-D and MST methods are gathered in Table 1.

**TABLE 1:** QCM-D and MST analyses of protein complex formation.

Enzyme complex	Kd [µM] QCM-D	Kd [µM] MST
TS/DHFR^a^	7.6 ± 0.7	2.8 ± 0.4
SHMT1/TS	No interactions	No interactions
SHMT1/pTS	5.35 ± 0.87	0.219 ± 0.02
SHMT1/DHFR	12.11 ± 7.86	12.60 ± 0.66
SHMT1/DHFR/TS	NA	5.04 ± 0.5
SHMT1/DHFR/pTS	0.28 ± 0.23	0.32 ± 0.05

a Antosiewicz et al., 2015; NA, not analyzed.

## Discussion

There are reports indicating the occurrence of CK2-mediated phosphorylation of TS, DHFR, and SHMT (Frączyk et al., 2010; Frączyk et al., 2015; Ludwiczak et al., 2016; Rusin et al., 2017; Skierka et al., 2019); however, to this date, there have been no studies showing the physiological role of CK2-mediated phosphorylation of thymidylate synthesis cycle enzymes. Our previous (Wińska et al., 2019) and present qPCR results indicate that inhibition of CK2 in CCRF-CEM cells treated with a specific CX-4945 inhibitor does not significantly influence the mRNA levels. The only statistically significant results showing some changes are the increase in DHFR (Wińska et al., 2019) and SHMT1 transcripts in cells treated with 6 μM CX-4945 for 24 h, decrease in TS and SHMT2 in cells treated with 3 μM CX-4945 for 72 h, and decrease in DHFR in cells treated with 6 μM inhibitor for 72 h. In each case, the change was well below 2-fold, so it can hardly be considered meaningful. Therefore, the intracellular increase of DHFR and SHMT1 protein levels upon CK2 inhibitor treatment may be attributed to the disturbance of some regulatory roles of CK2. Regarding this discrepancy between the mRNA and protein level of enzymes involved in the thymidylate biosynthesis cycle in cells after CX-4945 treatment, we concluded that CK2-mediated phosphorylation affects their stability in cells. Both DHFR and SHMT1 have been shown to undergo polyubiquitination and degradation in proteasome (Johnston et al., 1995; Anderson et al., 2012); moreover, proteasome inhibition stabilizes SHMT1 and increases ubiquitinated SHMT1 levels in a cell. It has been shown that the stability and localization of SHMT1 is regulated by competitive modification by Ubc-9 and Ubc-13. The former triggers SHMT1 nuclear degradation, whereas the latter increases SHMT1 stability (Anderson et al., 2012). Since there are examples for targeting CK2-phosphorylated proteins for ubiquitination and degradation, it cannot be excluded that the inhibition of CK2 may lead to the decrease of ubiquitination and, subsequently, the decrease of DHFR and SHMT1 as observed by us increased the levels of both proteins.

Interestingly, our results indicate that CK2 inhibition has a different impact on TS level compared with DHFR and SHMT1 protein levels. Our western experiments show that, in CCRF-CEM cells treated with the CK2 inhibitor, TS protein level decreases with the increase in concentrations of CX-4945, which is in contrast to DHFR and SHMT1 behavior. Considering that TS is a non-ubiquitinated proteasome substrate and undergoes only ubiquitin-independent proteasomal degradation (Erales and Coffino, 2014), the obtained results may confirm the possible role of CK2-mediated phosphorylation in the regulation of the ubiquitin-dependent degradation of DHFR and SHMT1.

Taking into account that DHFR is an important molecular target in anti-cancer therapy and CX-4945 is a potential anti-cancer agent, the significant elevation of DHFR concentration and its resulting activity increase in cells may contribute to the vast reduction in the effectiveness of treatment. There is a lot of evidence showing that the increased DHFR level makes the therapy ineffective. Among the drug resistance mechanisms in DHFR-targeted anti-cancer therapy, the best known is the amplification of the DHFR gene which leads to the increase in DHFR level after a prolonged methotrexate MTX treatment (Carman et al., 1984; Banerjee et al., 2002). In addition, since DHFR protein binds its own mRNA when its active site is unoccupied and, thereby, autoregulates its translation, binding of MTX to DHFR inhibits DHFR activity and also disrupts the interaction between DHFR and its own mRNA, relieving the repressed DHFR translation and leading to increased DHFR protein levels and, thus, to MTX resistance (Johnston et al., 1995). Considering that CX-4945 is a potential anti-cancer therapeutic agent, in view of our data showing the inhibition of CK2 activity causes the increase of DHFR and SHMT1 protein levels, a possible increased survival of cancer cells should be taken into consideration. However, further studies are needed to determine if prolonged inhibition of CK2 will not lead to resistance to cancer cells *via* increasing the level of thymidylate biosynthesis cycle enzymes.

The obtained results indicate that CK2-mediated phosphorylation of enzymes involved in the thymidylate biosynthesis cycle can be important for not only their stability within the cells but also for their distribution and ability to form complexes. Our studies involving immunodetection of DHFR and TS within A-549 cells confirmed that both protein levels and localizations in control cells are cell cycle dependent. It is known that TS mRNA level increases up to 20-fold in the S-phase (Navalgund et al., 1980), which is driven by the E2F transcription factor (DeGregori et al., 1995). On the other hand, the transcription of DHFR in the S-phase is driven by the E2F transcription factor working in concert with the Sp1 transcription factor (Abali et al., 2008). The decrease of immunodetection of both enzymes in untreated cells during incubation corresponds to the exit of the cells from the S phase. The obtained results indicate that CX-4945 treatment leads to the prolongation of the G2/M phase, which is in good agreement with the studies demonstrating the effect of CX-4945 on the cell cycle progression in different cell lines (Gray et al., 2014; Wińska et al., 2018). Moreover, the TS immunodetection results obtained in A-549 cells treated with CX-4945 correspond well to the western blot data for the same line, demonstrating the highest increase of TS protein level in cells treated with 0.5 IC_50_ CX-4945 and the decrease of this level at 1.5 IC_50_. It is worth noting that the significant decrease of TS in A-549 at IC_50_ and 1.5 IC_50_ CX-4945 corresponds to G2/M arrest in the cells. Interestingly, an immunodetection of DHFR in A-549 cells partially corresponds with the western blot data, since an increase in DHFR is detected in all CX-4945-treated cells in comparison to the control cells after 48 h of treatment; however, a decrease in the DHFR level in A-549 treated with the highest concentration of CX-4945 corresponding to the western blot data is detected after 72 h.

Using two methods, QCM-D and MST, we have shown the *in vitro* interactions of TS, SHMT1, and DHFR. In the QCM-D method, one protein is immobilized while the other serves as a ligand, whereas MST allows protein–protein interaction measurement with two or three proteins free in solution. Our results clearly show that TS interacts with DHFR and DHFR interacts with SHMT1; however, TS does not interact with SHMT1. Interestingly, all three proteins present in the solution interact and form a tri-complex measured with the MST method. Phosphorylation of TS by CK2 enables the interaction of the enzyme with SHMT1, and the tri-complex of pTS with SHMT1 and DHFR is more stable than the complex with non-phosphorylated TS. The obtained results suggest that phosphorylation of TS may act as a molecular switch, initiating a tri-enzymatic complex formation.

Taking into account that CK2 is a pleiotropic kinase and its inhibition affects different signaling pathways within the cells, the effects we detect do not have a simple explanation. However, considering that enzymes involved in the thymidylate biosynthesis cycle are key members of proliferation machinery, whereas CK2 is a potential anti-cancer molecular target, the obtained results may be relevant in the development of new anti-cancer therapies, based on CK2 inhibition. It should be taken into account that CK2 inhibition may affect folate and thymidylate metabolism in cells contributing to cell resistance to such therapies. Therefore, further detailed studies on the impact of CK2 inhibition on enzymes involved in the thymidylate biosynthesis cycle should be conducted.

## Conclusions

Many studies demonstrated that phosphorylation can affect the stability, the activity, and even the location of a protein within the cell. Our results suggest the potential regulatory role of CK2-mediated phosphorylation of enzymes involved in the thymidylate biosynthesis cycle in their stability, nuclear location, and ability to complex formation. Considering that CK2 is a potential anti-cancer target, whereas both TS and DHFR are well-established targets in chemotherapy, the obtained results seem to be important in developing new anti-cancer strategies. Therefore, further in-depth research should be carried out in order to clarify the physiological role of CK2-mediated phosphorylation of TS/DHFR/SHMT.

## Data Availability

The original contributions presented in the study are included in the article/Supplementary Material, further inquiries can be directed to the corresponding authors.
