# Supplementary material for: Inhibition of Protein Kinase CK2 Affects Thymidylate Synthesis Cycle Enzyme Level and Distribution in Human Cancer Cells
Source: Front Mol Biosci. 2022 Feb 25;9:847829. doi: 10.3389/fmolb.2022.847829 (PMC8914513; doi:10.3389/fmolb.2022.847829)
Supplement: Supplementary file 1 [file DataSheet1.DOCX]

Supplementary Material

Graphical presentation of QCM and MST methods


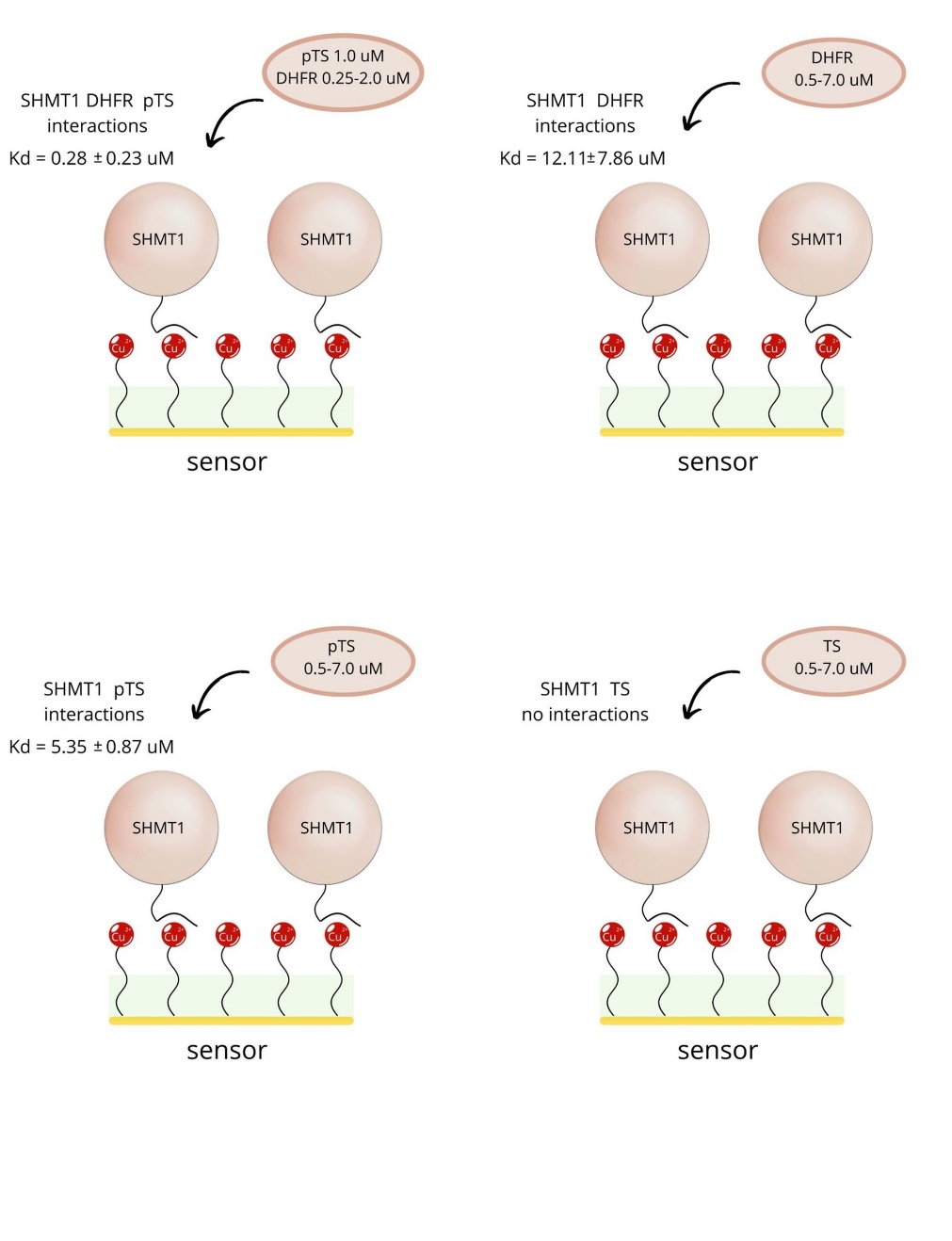


**Figure S1.** Protein immobilization on a sensor of QCM-D with a surface modified to catch His-tags. His-Tag-SHMT1 was immobilized on the sensor while TS, pTS, DHFR or both pTS and DHFR were introduced into the sensing chamber in solution at indicated concentration ranges.


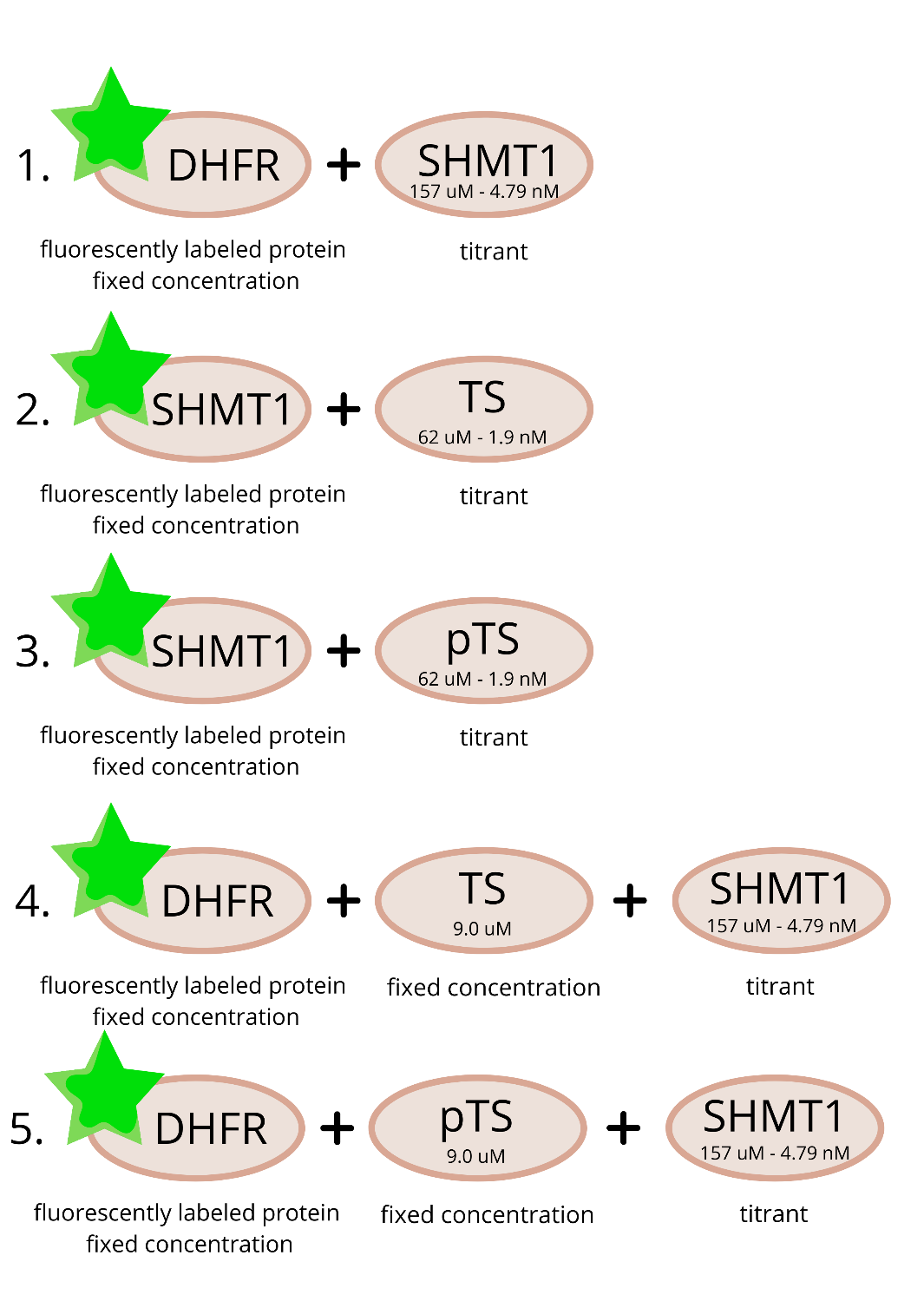


**Figure S2.** MST assay of specific interactions between proteins. DHFR or SHMT1 were fluorescently labeled. In two-protein interaction assay the unlabeled protein was a titrant added at various concentrations. In three-protein interaction assay one unlabeled protein was added at fixed concentration whereas the other served as a titrant.

**Effect of CX-4945 on viability of A-549 and CCRF-CEM**

MTT-based assay was used to determine IC50 values for CX-4945 for A-549 and CCRF-CEM. IC50 values are 4 and 31 µM for CCRF-CEM and A-549, respectively.

**Figure S3.** Viability of A-549 and CCRF-CEM treated with CX-4945 for 48 h. Dose response curves were generated in GraphPad Prism after fitting MTT-based assay data to the following equation: Y=Bottom + (Top-Bottom)/(1+10^((LogIC50-X)*HillSlope)).

**The *in vitro* complex formation studies showing the effect of TS phosphorylation.**

The interaction between SHMT1 and TS or SHMT1 and pTS were investigated using QCM-D and MST or QCM-D, respectively

**
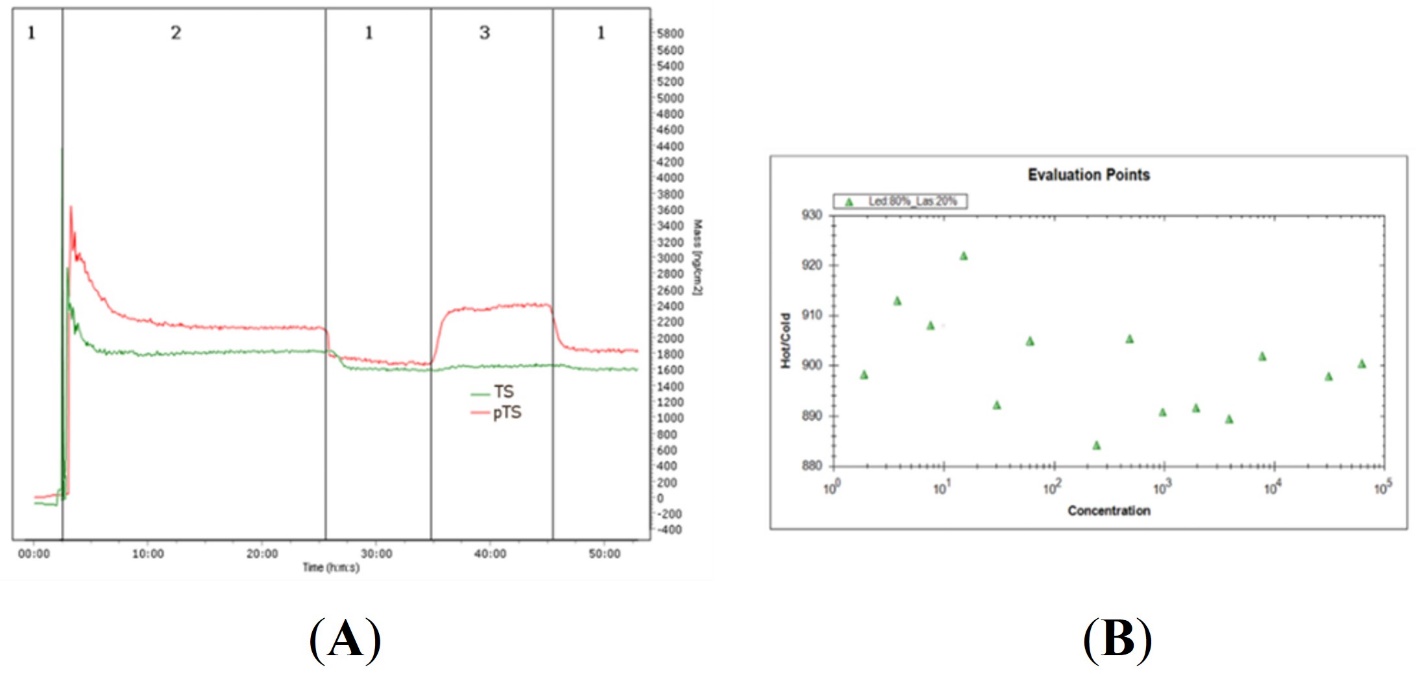
**

**Figure S4**. Lack of interaction between SHMT1 and non-phosphorylated TS studied by means of QCM-D (**A**) and MST (**B**). (**A**) The dependence of weight change on QCM-D sensor on time: 1. Washing the system with a buffer, 2. Immobilizing of 5 μM SHMT1 on the sensor surface, 3. Introducing of 1 μM TS or pTS. (**B**) SHMT1-TS interaction was measured using fixed concentration of labeled SHMT1 and various TS concentrations in the range of 62.0 μM – 1.9 nM.

**The effect of CK2-mediated TS phosphorylation on the formation of di- and tri-protein thymidylate cycle enzymes complexes**


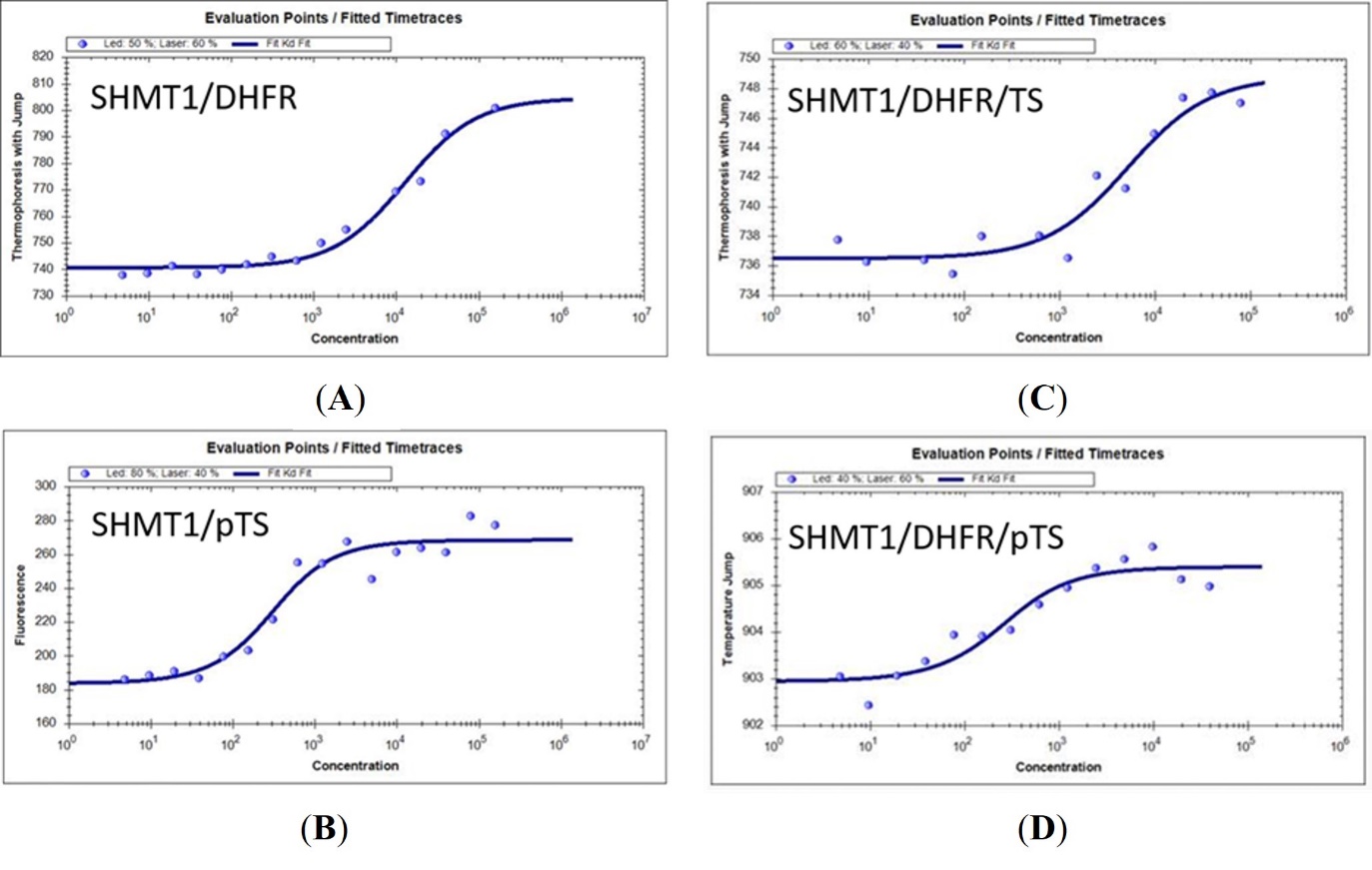


**Figure S5**. The study of protein-protein interaction by means of microthermoforesis. The graphs show the temperature induced fluorescent change as a function of the titrant concentrations. The dissociation constant Kd is obtained by fitting the binding curve with the quadratic solution for the fraction of fluorescent molecules that formed the complex, calculated form the law of mass action. We used DI.Screening Analysis software (NanoTemper) to fit the Kd model which allows to determine the strenght of molecular interactions occuring with either 1:1 stoichiometry or where several molecules A bind to one molecule B independently i.e. with no cooperativity. (**A**) The SHMT1-DHFR interaction was measured using fixed concentration of fluorescently labeled DHFR protein and various concentrations of SHMT1 in the range of 157 μM – 4.79 nM. (**B**) The SHMT1-pTS interaction measured in a sample containing fixed concentration of fluorescently labeled SHMT1 and various pTS titrant concentrations (157 μM – 4.79 nM). (**C**) and (**D**) For tri-complex formation assay samples contained constant concentrations of labeled DHFR, unlabeled TS or pTS (9.0 μM), and unlabeled SHMT1 titrant concentration in the range of 157 μM – 4.79 nM.
